# Variations in Outcomes of Surgical Oncology Fellowship Graduates Performing Hepatopancreatic Surgery in the United States Based on Fellowship Training Program

**DOI:** 10.1245/s10434-025-17587-0

**Published:** 2025-06-14

**Authors:** Diamantis I. Tsilimigras, Selamawit Woldesenbet, Odysseas P. Chatzipanagiotou, Valerie P. Grignol, Carlo M. Contreras, Jordan M. Cloyd, Timothy M. Pawlik

**Affiliations:** 1 Department of Surgery, Division of Surgical Oncology, The Ohio State University Wexner Medical Center and James Comprehensive Cancer Center, Columbus, OH USA; 2 Department of Surgery, The Urban Meyer III and Shelley Meyer Chair for Cancer Research, The Ohio State University, Wexner Medical Center, Columbus, OH USA

**Keywords:** Variations, Outcomes, HPB, Hepatopancreatic, Surgical oncologist, Fellowship, Program

## Abstract

**Background:**

Exposure in hepatopancreatic (HP) surgery can vary among Complex General Surgical Oncology (CGSO) fellowship graduates. Real-world outcomes of fellowship graduates performing HP surgery have not been previously examined.

**Methods:**

Medicare beneficiaries undergoing HP surgery for cancer between 2016 and 2021 were identified. Surgeon-level data, including fellowship training information, were linked to patient-level Medicare data. Trends and variations in severe complications and 90-day mortality according to fellowship training were examined.

**Results:**

Overall, 9954 HP cancer operations (pancreatectomy: 7,566, 76%; hepatectomy: 2,388, 24%) were performed between 2016 and 2021. A total of 609 CGSO fellowship graduates trained at 42 different CGSO programs in the United States or Canada were identified. Most cases (93.2%) were performed by surgeons who had completed an ACGME-accredited CGSO program. Almost half of HP operations were performed by graduates of two specific CGSO programs (*n *= 4,769, 47.9%), whereas 92.1% (*n *= 9,166) of HP operations were performed by graduates of 15 CGSO programs. After adjusting for relevant, multilevel characteristics, marked variations in outcomes by CGSO fellowship program were noted following both hepatectomy and pancreatectomy. The adjusted probability of serious complications decreased from 2016 to 2021 (16.4% vs. 12.9%; *p* < 0.05), however, the likelihood of 90-day mortality remained relatively stable during the study period (2016: 6.4% vs. 2021: 5.3%; *p* = 0.19).

**Conclusions:**

While outcomes of CGSO graduates improved over time, a marked variation in outcomes of graduates performing HP surgery was noted based on their fellowship training. Further efforts should be made to enhance and standardize HP surgery exposure and training in CGSO programs for fellows intending to perform HP surgery in practice.

Hepatopancreatic (HP) surgery is a highly complex field that includes technically challenging operations associated with considerable morbidity and mortality.^1–3^ Advanced, well-structured training is required to achieve proficiency with these operations and optimize patient outcomes.^3,4^ Complex General Surgical Oncology (CGSO) fellowships, which are accredited by the Accreditation Council for Graduate Medical Education (ACGME), aim to prepare surgeons with the necessary knowledge and skills to treat multiple cancers, including HP malignancies.^5^ Despite the standard curriculum of CGSO fellowship training, there remains marked variability in operative exposure and experience, particularly regarding HP operations, across different fellowship programs.^3,4^

Previous studies have examined the role of surgeon volume, career stage, and hospital resources relative to outcomes following HP surgery.^6–9^ Nevertheless, little is currently known about the impact of CGSO fellowship program training on outcomes of patients undergoing HP surgery for cancer by their graduates. Differences in training paradigms, case complexity, operative autonomy, mentorship, and institutional resources may contribute to variations in surgeon proficiency at the time of fellowship graduation and may, in turn, influence patient outcomes after HP surgery.^6,10^ Understanding variations in patient outcomes after HP surgery performed by graduates of different fellowship programs is critical to identify gaps in training, improve surgical education, and optimize patient care. To this end, the objective of the current study was to evaluate real-world outcomes among CGSO fellowship graduates performing HP surgery for cancer in the United States. In particular, using a nationwide cohort of Medicare beneficiaries, this study sought to assess variations in severe complications and 90-day mortality across surgeons from different CGSO fellowship programs. By identifying variations in outcomes by fellowship training program, the findings may inform efforts to standardize HP surgical training to ensure surgical competency and patient safety across all training programs.

## Methods

### Study Population and Databases Used

The data were obtained from the 100% Medicare Standard Analytic Files provided by the Centers for Medicare and Medicaid Services (CMS) for fee-for-service beneficiaries enrolled in Medicare Part A and B. The study included patients aged 65 years and older who underwent HP surgery for cancer between 2016 and 2021. Relevant HP operations were identified using International Classification of Diseases, Tenth (ICD-10) Procedure Codes, as previously described.^6^ Exclusion criteria included: no enrollment in Medicare parts A and B during the month of the HP surgery, receipt of additional payments from a Health Maintenance Organization, patient age younger than 65 years and surgery for benign indications.

To incorporate hospital-level data into the current analysis, data from the American Hospital Association (AHA) database were also merged to patient-level Medicare data. Hospital-level data of interest included intensive care unit (ICU) availability, institution teaching status, nurse-to-bed ratio as well as number of beds. Teaching status in the AHA database may refer to non-university affiliated institutions. A novel database including data on surgeon fellowship training and year of graduation was built, as previously described.^6^ In short, two independent reviewers manually screened all National Provider Identification (NPI) numbers associated with surgeons who performed the procedures of interest in Medicare database. Publicly available sources including the American Board of Surgery (ABS) as well as institutional/hospital websites were screened to extract surgeon-level data on fellowship program attended and year of fellowship graduation.^6^ Discrepancies in data extraction were resolved after re-reviewing discordant data and after reaching consensus among independent reviewers. The current study only analyzed procedures performed by surgeons who had completed a surgical oncology (SONC) (before ACGME accreditation) or CGSO (after ACGME accreditation) fellowship. Surgeons who had not completed a SONC/CGSO fellowship or had inadequate / missing information were excluded. Surgeon-level data were subsequently linked to patient-level Medicare data.

### Variables and Outcomes of Interest

Variables that were accounted for in the current study included patient-level data (i.e., patient age, sex, race, social vulnerability index [SVI], and Charlson score), procedure-related data (i.e., type of surgical procedure [minor/major hepatectomy, pancreatectomy (non-Whipple), Whipple], year of surgery, admission type [elective vs. urgent]), hospital-level characteristics (i.e., teaching status, intensive care unit availability, rurality, nurse-to-bed ratio, and number of hospital beds). In addition, the current study accounted for surgeon-level characteristics, including surgeon sex, case-specific volume (i.e., meeting Leapfrog standards),^11^ years of experience after fellowship graduation, and graduating fellowship program. Surgeons were also categorized according to their career stage as previously described^12^; early (1–7th year of independent practice), middle (8–14th year), or late career stage (≥15th year). To avoid outliers, surgeons who were in practice for more than 30 years were excluded from the study.

Outcomes of interest included serious complications and 90-day mortality. Serious complications were defined as the occurrence of a complication combined with prolonged length of hospital stay (>75th percentile), as previously described.^12^ A validated list of postoperative complications was assessed along with the addition of liver-specific (e.g., bile duct injury, bile leak) and pancreatic-specific complications (e.g., pancreatic leak/fistula and delayed gastric emptying).^13–15^ If a complication was present on admission, it was excluded from the definition of index complication, as previously described.^12^ Mortality was defined as any death occurring within 90 days after the index surgery. The current study was deemed exempt by the Institutional Review Board of the Ohio State University, as all data were de-identified.

### Statistical Analysis

Median values (interquartile range [IQR]) and frequency (%) were used to describe continuous and categorical variables, respectively. Multivariable logistic regression analysis was performed to calculate the adjusted probability of serious complications and 90-day mortality among CGSO fellowship programs after adjusting for patient-level (i.e., age, sex, race, SVI, Charlson score), procedure-level (i.e., year of surgery, type and complexity of surgery, admission type), hospital-level (i.e., hospital rurality, teaching status, nurse-to-bed ratio), and surgeon-level (i.e., surgeon sex, surgeon volume, and fellowship program) characteristics. Due to the presence of >40 different fellowship programs and to avoid outliers, adjusted probabilities of serious complications and 90-day mortality were calculated relative to the top 15 fellowship programs, which were selected based on the total number of HP cases performed by their graduates in the Medicare dataset during the study period (92.1% of all cases). This selection reflected case volume of graduates and did not imply a qualitative ranking of the programs. The Wald test was performed to assess whether the estimated probabilities for certain fellowship programs differed significantly. The Wald test was used to evaluate the null hypothesis that the adjusted odds of serious complications and 90-day mortality were equal between program X and program Y. A subgroup analysis was performed to assess variations in outcomes among early career surgical oncologists only. All statistical tests were two-tailed with statistical significance set at α = 0.05. All data analyses were conducted by using STATA (version 18.0, StataCorp, College Station, TX).

## Results

### Cohort Characteristics

A total of 9,954 patients underwent HP surgery for cancer during the study period (Table 1). Median patient age was 72 years (interquartile range [IQR] 69–77); the majority of patients was White (*n *= 8,794, 88.3%) and male (*n *= 5,380, 54%). Most patients underwent pancreatectomy (Whipple: *n *= 5,142, 51.7%; non-Whipple pancreatectomy: *n *= 2,424, 24.4%), whereas one fourth underwent hepatectomy (minor hepatectomy: *n *= 1,762, 17.7%; major hepatectomy: *n* = 626, 6.3%) for a malignant indication. Most patients underwent surgery at a teaching hospital (*n *= 7,348, 73.8%) with more than 500 beds (*n *= 7,606, 76.7%) located in a metropolitan area (*n *= 8,185, 82.4%).

**Table 1:** Patient, hospital, and surgeon characteristics of patients undergoing hepatopancreatic surgery for cancer by all surgical oncologists and early career surgical oncologists

	Total cohort (*n* = 9,954)	Treated by early-career surgical oncologists (*n* = 3,326)
Patient characteristics*		
Age, years (IQR)	72 (69–77)	72 (68–77)
*Patient sex*		
Female	4,574 (46.0%)	1,541 (46.3%)
Male	5,380 (54.0%)	1,785 (53.7%)
*Race*		
White	8,794 (88.3%)	2,911 (87.5%)
Non-White	1,160 (11.7%)	415 (12.5%)
*SVI*		
Low	3,446 (34.7%)	1,121 (33.8%)
Medium	3,388 (34.1%)	1,151 (34.7%)
High	3,095 (31.2%)	1,048 (31.6%)
Charlson score	3 (2-8)	3 (2-8)
*Year of surgery*		
2016	1,487 (14.9%)	504 (15.2%)
2017	1,611 (16.2%)	529 (15.9%)
2018	1,697 (17.0%)	578 (17.4%)
2019	1,749 (17.6%)	586 (17.6%)
2020	1,700 (17.1%)	555 (16.7%)
2021	1,710 (17.2%)	574 (17.3%)
*Procedure type*		
Pancreatectomy	7,566 (76.0%)	2,495 (75.0%)
Hepatectomy	2,388 (24.0%)	831 (25.0%)
*Case complexity*		
Minor hepatectomy	1,762 (17.7%)	635 (19.1%)
Major hepatectomy	626 (6.3%)	196 (5.9%)
Pancreatectomy (non-Whipple)	2,424 (24.4%)	764 (23.0%)
Whipple	5,142 (51.7%)	1,731 (52.0%)
*Admission type*		
Elective	9,352 (94.0%)	3,125 (94.0%)
Urgent	599 (6.0%)	201 (6.0%)
Hospital characteristics*		
*Rurality*		
Urban	1,744 (17.6%)	653 (19.7%)
Metropolitan	8,185 (82.4%)	2,667 (80.3%)
*Teaching status*		
No	2,606 (26.2%)	1,014 (30.5%)
Yes	7,348 (73.8%)	2,312 (69.5%)
ICU availability	9,339 (99.6%)	3,130 (99.1%)
Nurse-to-bed ratio	2.2 (1.6-3.3)	2.2 (1.6-3.3)
Number of beds >500	7,606 (76.7%)	2,488 (75.0%)
Surgeon characteristics*		
*Surgeon sex*		
Female	1,030 (10.3%)	545 (16.4%)
Male	8,924 (89.7%)	2,781 (83.6%)
*Surgeon volume*		
Low	5,145 (51.7%)	1,976 (59.4%)
High	4,809 (48.3%)	1,350 (40.6%)
Years of experience, mean (standard deviation)	11.764 (7.304)	4.084 (2.021)
Early career	3,326 (33.4%)	3,326 (100%)
Middle career	3,379 (33.9%)	–
Late career	3,249 (32.6%)	–
*CGSO + additional fellowship*		
No	9,390 (94.3%)	3,110 (93.5%)
Yes	564 (5.7%)	216 (6.5%)

^*^Patient-level data

*SVI* social vulnerability index; *ICU* intensive care unit; *CGSO* complex general surgical oncology

### Surgeon and Fellowship Program Characteristics

During the study period, a total of 609 surgical oncologists who trained at 42 different CGSO/SONC programs (both accredited and nonaccredited programs) in the United States or Canada performed all HP operations were identified. Most operative cases (93.2%) were performed by surgeons who had completed an ACGME-accredited CGSO program (based on current accreditation status). Among all HP cases, 33.4% (*n *= 3,326) were performed by early career surgical oncologists, 33.9% (*n *= 3,379) by middle career surgical oncologists and 32.6% (*n *= 3,249) by late career surgical oncologists. Of note, almost one-half of HP operations were performed by graduates of two specific CGSO programs (*n *= 4,769, 47.9%), whereas 92.1% (*n *= 9,166) of HP operations were performed by graduates of the 15 top CGSO programs (ranked by frequency of cases performed by graduates of these programs, not by reputation or other quality metrics).

### Variations in Serious Complications and 90-day Mortality by Fellowship Program

**Fig. 1:**
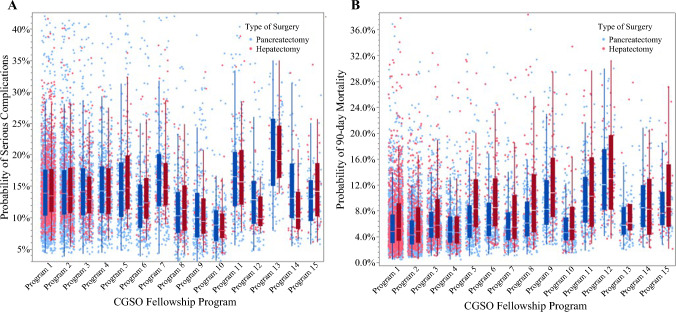
Adjusted probability of serious complications (a) and 90-day mortality (b) after hepatopancreatic surgery for cancer across all surgical oncologists relative to graduating CGSO fellowship program

**Fig. 2:**
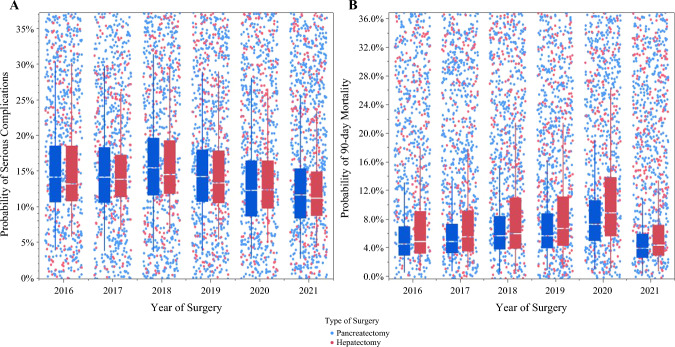
Trends in the adjusted probability of serious complications (a) and 90-day mortality (b) after hepatopancreatic surgery for cancer performed by all surgical oncologists from 2016 to 2021

**Fig. 3:**
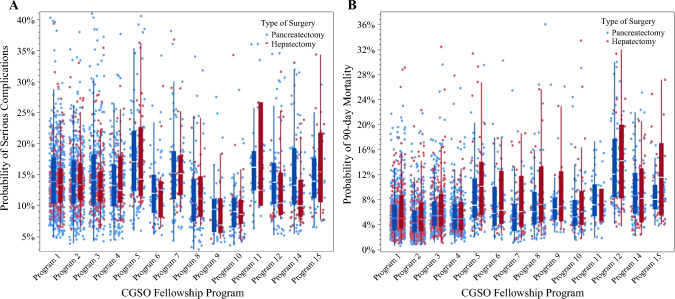
Adjusted probability of serious complications (a) and 90-day mortality (b) after hepatopancreatic surgery for cancer across early career surgical oncologists relative to graduating CGSO fellowship program

**Fig. 4:**
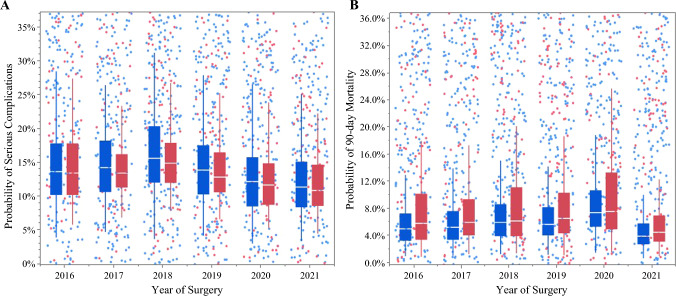
Trends in the adjusted probability of serious complications (a) and 90-day mortality (b) after hepatopancreatic surgery for cancer performed by early career surgical oncologists from 2016 to 2021

The overall incidence of serious complications and 90-day mortality following HP surgery for cancer in the entire cohort was 14.8% (*n *= 1,478) and 6.9% (*n *= 684), respectively. After controlling for patient-, procedure-, hospital-, and surgeon-level characteristics, marked variations in outcomes were noted by graduating CGSO fellowship program. For example, the risk-adjusted incidence of severe complications was 9.7% among graduates of CGSO program #10 versus 21.7% (%difference 12.0%) among graduates of CGSO program #13 following HP surgery (*p *= 0.001). Similarly, the risk-adjusted incidence of 90-day mortality was 5.5% among graduates of CGSO program #7 versus 12.1% (% difference 5.1%) among graduates of CGSO program #12 following HP surgery (*p *= 0.002) (Fig. 1). When trends in outcomes over time were assessed, the adjusted risk of serious complications decreased from 2016 to 2021 (16.4% vs. 12.9%; *p *< 0.05), yet the likelihood of 90-day mortality remained relatively stable during the study period (2016: 6.4% vs. 2021: 5.3%; *p* = 0.19) (Fig. 2). Similar trends were noted when assessing outcomes following HP cancer surgery performed by early-stage career surgical oncologists only (Figs. 3 and 4).

## Discussion

The training paradigm in HPB surgery has changed over the last several decades.^4,16^ While different fellowship pathways exist today that prepare surgeons for a HPB surgery career (i.e., CGSO, American Hepato-pancreato-biliary Association [AHPBA], and American Society of Transplant Surgeons [ASTS] fellowships),^16^ the vast majority of HP surgeries in the United States are currently performed by CGSO fellowship graduates.^6^ Nevertheless, real-world data to inform quality of HP training among graduates of different CGSO fellowship programs have not been available to date. This study specifically assessed outcomes of patients following HP cancer surgery relative to the fellowship program completed by their treating surgeon. The data demonstrated marked variations in severe complications and 90-day mortality based on fellowship training program after adjusting for relevant patient, procedure, hospital, and surgeon-level characteristics. To our knowledge, this study represents the first, large-scale, real-world analysis of outcomes among CGSO/SONC fellowship graduates performing HP surgery in the United States.

The CGSO fellowship pathway is designed to provide broad exposure to complex oncologic surgery, including endocrine, breast, soft tissue, sarcoma, HPB, and other non-HPB gastrointestinal operations.^5,17^ Currently, there are 35 ACGME-accredited CGSO fellowship programs in North America.^18^ The minimum HPB case requirements for a graduating CGSO fellow include 35 operative HPB cases (out of the total 170 required oncologic cases) and 25 HPB multidisciplinary cases (out of total 120 required cases).^17^ Although the minimum case numbers are universal across all programs, operative experience in HP surgery may vary across different institutions. Although data on case volume during training were not available for further analysis in the context of this study, it was notable that nearly half of all HP procedures in the current cohort were performed by graduates of just two CGSO fellowship programs, while 92.1% of all HP cases were performed by graduates of the top 15 programs (ranked by frequency of cases performed by graduates) (out of the 42 programs in total). Previous studies have reported that graduates of certain CGSO fellowship programs may feel more comfortable performing HP operations than others. In a survey of CGSO graduates from 2012 to 2022, approximately 15% of respondents felt clinically unprepared for hepatobiliary practice and 24% technically unprepared for hepatobiliary surgery.^19^ Although all programs adhere to the same case requirements, certain programs may consistently produce surgeons with a HPB focus, while others might offer more focused training in different oncologic subspecialties (i.e., breast, melanoma/sarcoma, etc.). As such, carefully selecting a program that aligns with a fellow’s career aspirations and future clinical practice is critical to ensure adequate training, particularly in technically challenging fields, such as HPB surgery.

Marked variation in outcomes was observed even among graduates of the top 15 CGSO fellowship programs (ranked by frequency of cases performed by graduates of each program). Importantly, this variation was also evident among early career surgeons (i.e., surgeons within 1–7 years after fellowship graduation), highlighting the importance of robust training in HP surgery to prepare fellows for independent practice. Although the reasons behind this variation may be multifactorial, possible explanations include differences in operative autonomy and total number of HP cases performed during fellowship training, quality of mentorship, and whether or not certain programs offer enhanced HPB training during fellowship. To date, 8 of the 35 ACGME-accredited CGSO fellowship programs provide dual certification in both CGSO and HPB surgery (via AHPBA certification),^20^ which enhances operative exposure in HP surgery and improves surgical proficiency for HPB designated fellows. Indeed, requirements for an AHPBA certificate currently include a minimum of 48 weeks of clinical training focused on HPB patients with a total of 100 HPB cases, of which at least 20 represent major hepatectomies and 25 pancreatoduodenectomies.^20^ Not many programs have the ability to achieve these numbers and provide dual certification for fellows interested in a career in HPB surgery. In a highly specialized and technically challenging field, such as HPB surgery, differences in training experience and operative volume may be associated differences in patient outcomes.^6^ The findings of this study complement prior work from our group that evaluated outcomes after HP surgery by fellowship track (CGSO, AHPBA, transplant).^6^ Although the CGSO fellowship remains the predominant training pathway for surgeons performing HP surgery in the United States, a substantial variation exists in HP-related outcomes by CGSO fellowship program.^6^ Although the overall 90-day mortality following HP surgery for cancer was consistent with what has been reported in previous Medicare-based studies,^7,14,21^ mortality after HP surgery varied among graduates from different programs. Monitoring performance metrics of different fellowship graduates can help identify gaps in training and inform curriculum improvements. Further efforts are needed to optimize HP-related training curriculum within CGSO fellowship pathway and standardize HP training across different programs to improve proficiency of fellows aiming to practice HP surgery after fellowship graduation.

One of the main strengths of the current study is that it was the first to assess outcomes of surgical oncologists based on the CGSO/SONC fellowship program completed. Since there is currently no database that includes surgeon-level data regarding year of fellowship graduation and fellowship program attended, these data were manually extracted for each individual surgeon and linked the information to patient-level Medicare data. This unique approach allowed us to assess real-world data for surgical oncologists performing HP cancer surgery in the United States for the first time. Nevertheless, certain limitations should be taken into account when interpreting the results of the current study. Although surgeons who had completed an additional fellowship (apart from CGSO) were identified, assessing the potential impact of dual certification status (CGSO board certification and AHPBA certification) on patient outcomes was not feasible. In addition, this study focused solely on cancer cases limiting the generalizability of findings to benign HP diseases. Furthermore, as with all billing databases, the possibility of miscoding or variations in coding practices cannot be ruled out. Nevertheless, a previously validated list of complications was used to ensure high fidelity.^6,13–15^ Because the study population consisted of individuals aged 65 years and older, the findings may not be generalizable to younger individuals or individuals not enrolled in Medicare.

## Conclusions

While outcomes of CGSO/SONC graduates improved over time, a marked variation in outcomes among graduates performing HP surgery was noted based on fellowship training. Further efforts should be made to enhance and standardize HP surgery exposure and training in CGSO programs for fellows intending to perform HP surgery in their practice.
